# Current and future genomic applications for surgeons

**DOI:** 10.1308/rcsann.2024.0031

**Published:** 2024-04-01

**Authors:** O Alkhatib, T Miles, RP Jones, R Mair, R Palmer, H Winter, FD McDermott

**Affiliations:** ^1^ Liverpool University Teaching Hospitals NHS Foundation Trust, UK; ^2^ Southwest Genomics Medicine Service Alliance, UK; ^3^ Institute of Systems, Molecular and Integrative Biology, University of Liverpool, UK; ^4^ University of Cambridge, UK; ^5^ CRUK Cambridge Institute, University of Cambridge, UK; ^6^ North Bristol NHS Trust, UK; ^7^ University Hospitals Bristol and Weston NHS Foundation Trust, UK; ^8^ Royal Devon University Healthcare NHS Foundation Trust, UK

**Keywords:** genetics, genomics, cancer vaccine

## Abstract

Genomics is a crucial part of managing surgical disease. This review focuses on some of the genomic advances that are available now and looks to the future of their application in surgical practice.

Whole-genome sequencing enables unbiased coverage across the entire human genome of approximately three billion base pairs. Newer technologies, such as those that permit long-read sequence analysis, provide additional information in longer phased fragment and base pair epigenomic (methylomic) data. Whole-genome sequencing is currently available in England for cancers in children, teenagers and young adults, central nervous system tumours, sarcoma and haematological malignancies.

Circulating tumour DNA (ctDNA), immunotherapy and pharmacogenomics have emerged as groundbreaking approaches in the field of cancer treatment. These are now revolutionising the way oncologists and surgeons approach curative cancer surgery.

Cancer vaccines offer an innovative approach to reducing recurrence after surgery by priming the immune system to trigger an immune response. The Cancer Vaccine Launch Pad project facilitates cancer vaccine studies in England. The BNT122-01 trial is recruiting patients with ctDNA-positive high-risk colorectal cancer after surgery to assess the impact of cancer vaccines.

The evolving landscape of cancer treatment demands a dynamic and integrated approach from the surgical multidisciplinary team. Immunotherapy, ctDNA, pharmacogenomics, vaccines, mainstreaming and whole-genome sequencing are just some of the innovations that have the potential to redefine the standards of care. The continued exploration of these innovative diagnostics and therapies, the genomic pathway evolution and their application in diverse cancer types highlights the transformative impact of precision medicine in surgery.

## Introduction

The Royal College of Surgeons of England Commission on the Future of Surgery highlighted areas including robotics, artificial intelligence and genomics for the evolution of surgery in the 21^st^ century.^1^ Over the past 50 years, the adoption of new healthcare technologies has improved patient care. With an increasing global population comprising older and more complex patients, there has never been a better time to maximise the benefits of new technologies. Surgeons and the wider surgical team manage a large disease burden in emergency and elective settings. Utilising genomics can assist in screening patients, improve diagnostics and treatments, and lead to better personalised care.^2^

This review describes some of the current and future applications of genomics in surgery, particularly in cancer care. This includes the use of circulating tumour DNA (ctDNA), immunotherapy, cancer vaccines, pharmacogenomics, next-generation sequencing and mainstream germline testing for patients by members of the multidisciplinary team. Genomics is crucial for current and future healthcare. However, there also needs to be engagement with surgeons and stakeholders to ensure that adequate support, education, training and evolution of curricula keep pace with these exciting developments.

## Circulating tumour DNA and immunotherapy

Immunotherapy has emerged as a groundbreaking approach in the field of cancer treatment,^3,4^ now revolutionising the way oncologists and surgeons approach curative cancer surgery.^5–7^ One significant aspect of immunotherapy involves understanding the molecular landscape of tumours. High microsatellite instability (MSI-high) is a genetic marker that indicates a higher likelihood of response to immunotherapy.^8^ Tumours with MSI-high status exhibit a greater number of genetic mutations, making them more susceptible to immune checkpoint inhibitors, a class of immunotherapy drugs that block inhibitory signals, enabling the immune system to recognise and attack cancer cells more effectively.

High tumour mutational burden (TMB-H) is seen as a potential predictor for checkpoint inhibitor response owing to its link to immunogenic neoantigens.^9,10^ The complex nature of immunotherapy response involves multiple factors and ongoing research aims to refine predictive markers. In clinical practice, the utility of TMB-H varies by cancer type. The evolving field emphasises the need for up-to-date information, continued consultation with healthcare professionals and communication with our patients.

In the realm of cancer genomics, ctDNA has become a valuable tool for surgeons and oncologists. ctDNA refers to fragments of tumour DNA circulating in the bloodstream, offering a non-invasive method to assess the genetic profile of a patient's cancer and detecting minimal residual disease. This information can guide treatment decisions, including the selection of targeted therapies or immunotherapies tailored to the specific genetic alterations present in the tumour. It may also help to select those patients at highest risk of relapse after surgery.^11^

In the context of improving outcomes from curative cancer surgery, the integration of cancer genomics is particularly crucial during neoadjuvant systemic anti-cancer therapy. Personalising the neoadjuvant therapy may optimise surgical resection, reduce risks of local and distant recurrence, and guide postoperative treatment and follow-up.

Lung cancer, a leading cause of cancer-related mortality worldwide, exemplifies the evolving backdrop of cancer treatment. Traditionally, chemotherapy had long been a cornerstone of lung cancer management but the advent of molecular profiling and immunotherapy has transformed the therapeutic landscape. Checkpoint inhibitors have demonstrated remarkable efficacy in certain subsets of lung cancer patients, particularly those with high PD-L1 expression. Neoadjuvant combination of chemotherapy with immunotherapy is now approved and being given to patients in the UK. Nevertheless, it is the molecular testing that has transformed lung cancer management. The testing of two driver mutations, *EGFR* mutations and *ALK* fusions, in patients with non-squamous non-small cell lung cancer has been embedded in guidelines for nearly a decade. However, the 2021 World Health Organization guidelines recommend testing for additional gene mutations for which targeted therapies are now available, including *ROS1*, *RET*, *NTRK1–3*, *KRAS*, *BRAF* and *MET*.^12^

The application of ctDNA extends beyond lung cancer to other challenging malignancies like cholangiocarcinoma. This bile duct cancer poses diagnostic and therapeutic challenges but ctDNA analysis provides a minimally invasive means of tracking genetic alterations and monitoring treatment response. Surgeons dealing with cholangiocarcinoma can benefit from the insights offered by ctDNA to tailor interventions based on the evolving genetic landscape of the disease, and tissue testing for *FGFR2*, *NTRK1–3*, *IDH1–2* and MSI is becoming mainstream.^13^

In cancer surgery, the integration of immunotherapy, chemotherapy and cancer genomics underscores the need for a multidisciplinary approach. Surgeons play a pivotal role in coordinating treatment strategies that optimise the chances of a curative outcome. By leveraging the power of immunotherapy, understanding MSI status, incorporating ctDNA analysis, and tailoring neoadjuvant and adjuvant therapies, surgeons can contribute to a paradigm shift in the management of malignancies. Multi-cancer early detection tests look at blood signals in those with and without symptoms, and offer hope for earlier opportunities for less invasive curative surgery.^14^

Adjuvant cancer vaccines represent another innovation against cancer.^15–17^ These vaccines aim to stimulate the immune system to recognise and attack residual cancer cells after surgery. By training the immune system to mount a sustained response against cancer-specific antigens, adjuvant cancer vaccines hold promise in preventing disease recurrence and improving long-term outcomes. A randomised phase 3 trial is underway across the UK in patients with high-risk colorectal cancer after surgery.^18^ Phase 1 data are emerging on the use of messenger RNA (mRNA) vaccines to stimulate T cells to recognise neoantigens in patients with pancreatic cancer.^19^ Anticipation is keenly focused on the delivery of larger randomised controlled trials across numerous tumour types.

## Cancer vaccines

Cancer vaccines offer an innovative approach to reducing recurrence after surgery by priming the immune system to trigger both an innate and an adaptive immune response. Different vaccine approaches are employed based on the cancer target. Some vaccines use oncolytic viruses that infect and kill target cancer cells directly.^20^ Others use antigens expressed by the tumour (with lower levels of expression in normal tissue) to prime the immune response. These responses include the development of tumour-infiltrating lymphocytes, which target residual cells expressing the antigen primed for by the vaccine.^18^

One major obstacle has been the lack of universal antigens expressed by all tumours as well as the expression of those same antigens in normal tissue. mRNA technology (which was used successfully in the development of COVID-19 vaccines) is an exciting development that allows patient-specific antigens to be identified and generated using novel next-generation sequencing and bioinformatic neoantigen prediction.

By introducing this exogenous synthetic mRNA into antigen-presenting cells, personalised tumour-specific antigens can be synthesised without immediate degradation by the immune system. These antigens are then delivered to the surface of these antigen-presenting cells via major histocompatibility complex molecules, triggering a rapid and durable expansion in T cell populations targeting these specific antigens. Crucially, mRNA vaccines are individualised by generating a bespoke vaccine from each sequenced tumour. These vaccines can be produced in a clinically meaningful timeframe (within 9 weeks of tumour sampling), flexibly and efficiently. mRNA is therefore a hugely promising platform for personalised neoantigen vaccine preparation.^19,21^

The most compelling clinical data to date around the efficacy of mRNA vaccines relate to their use as adjuvant therapy after resected pancreatic cancer. A phase 1 study from Memorial Sloan Kettering Cancer Center delivered adjuvant individualised mRNA vaccines alongside adjuvant chemotherapy after pancreatic resection.^19^ Sixteen patients received atezolizumab and an mRNA vaccine, and fifteen of these were also administered a modified FOLFIRINOX (folinic acid, fluorouracil, irinotecan, oxaliplatin) regimen. The vaccine triggered high-magnitude neoantigen-specific T cells in 8 of the 16 patients. At a median follow-up of 18 months, patients with vaccine-expanded T cells (responders) had no evidence of cancer recurrence whereas patients without vaccine-expanded T cells (non-responders) had a median recurrence-free survival of 13.4 months (*p*=0.003).

**Figure 1:**
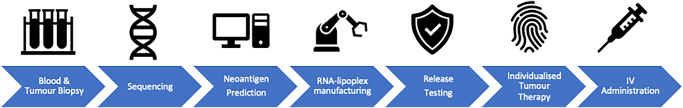
Simplified schematic of mRNA vaccine production

**Figure 2:**
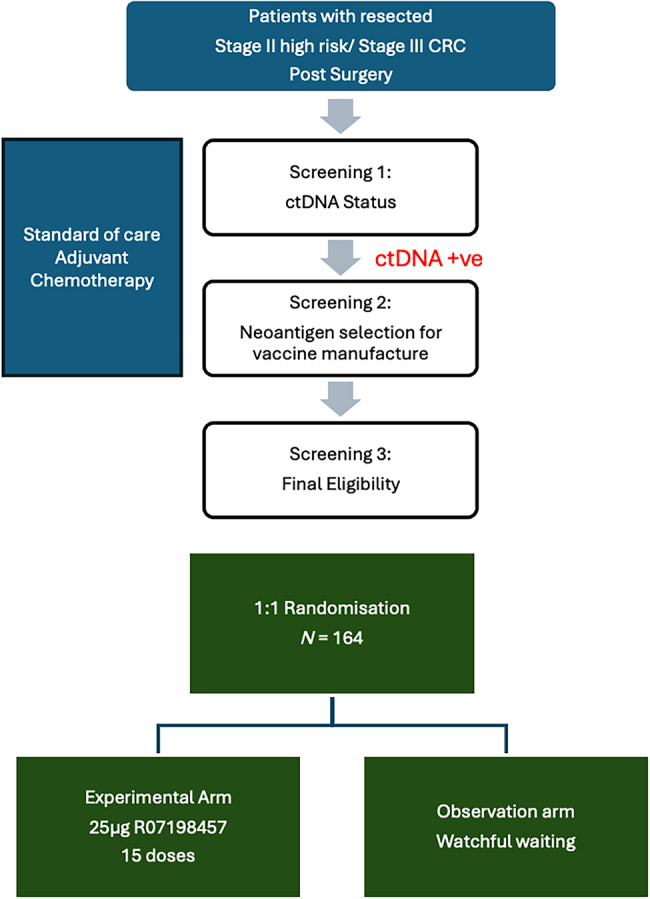
Simplified schematic of the BNT122-01 trial^18^ CRC = colorectal cancer; ctDNA +ve = circulating tumour DNA positive; R07198457 = experimental treatment/cancer vaccine

Owing to the low levels of preserved antigens between patients, colorectal cancer is another attractive setting for personalised mRNA vaccine technology (Figure 1). The BNT122-01 trial is the first of a series of cancer vaccine trials rolled out across the UK as part of a national partnership brokered by the Department of Health and Social Care, and the National Institute for Health and Care Research.^18^ The objective is to see whether the addition of individualised mRNA vaccines improves disease-free survival in high-risk patients with stage 2/3 R0 resected colorectal cancer who are ctDNA positive and being treated with adjuvant chemotherapy (Figure 2). ctDNA is a promising prognostic biomarker for early recurrence in colorectal cancer and selects a population at high risk of recurrence.^22,23^ BNT122-01 is a global study that is open in 14 UK sites and will randomise 164 patients. It is anticipated that the findings from the trial will inform the role of mRNA personalised cancer vaccines in resected stage 2/3 colorectal cancer and future guidelines for the management of these patients.^18^

## Pharmacogenomics

Pharmacogenomics describes how a patient’s response to a medicine may be influenced by an individual’s genomic make-up.^24^ Pharmacogenomic variants are common, found in approximately 98% of the population,^25^ potentially increasing susceptibility to adverse drug reactions or treatment inefficacy. *Accelerating Genomic Medicine in the NHS*, the five-year genomics strategy for England published in 2022, sets out the ambition to incorporate pharmacogenomics into routine practice to reduce adverse drug reactions.^26^ There are some tests that are now routinely available through NHS England’s national genomic test directory such as *DPYD* and clopidogrel for patients following ischaemic stroke.^13^ Although not currently part of routine practice, other pharmacogenomics tests have the potential to transform practice in a number of other areas.

### Dihydropyrimidine dehydrogenase (DPYD)

Three pharmacogenomic tests are commissioned in England via the national genomic test directory, including DPYD genotyping prior to fluoropyrimidine chemotherapy (e.g. capecitabine and fluorouracil).^13^ Roughly 3–5% of the population carry a *DPYD* variant,^27,28^ resulting in accumulation of fluoropyrimidines and severe or even fatal toxicity.^29^ *DPYD* genotyping for the four most common variants is now embedded into routine care.^30^ This is based on data from predominantly White Caucasian populations, and further research continues into variants in patients of non-European ancestry and rarer *DPYD* variants, with hopes of a more extensive genotyping panel in future.

### Clopidogrel and CYP2C19

Clopidogrel is a prodrug, converted to its active metabolite predominantly by CYP2C19. Around 20% of European ancestry and 40–45% of Asian ancestry populations carry one loss-of-function *CYP2C19* allele, with 2% of Europeans and 8–13% of Asians carrying two loss-of-function alleles.^31^ Data, predominantly from acute coronary syndrome (ACS) and also stroke indications, link the presence of *CYP2C19* loss-of-function(s) to poorer outcomes.^31^ Data are less well established for non-ACS cardiovascular indications^32^ but research continues in this area.^33^

### Analgesia

Approximately 25% of individuals may exhibit a lack of response to codeine or tramadol and/or toxicity due to variants in the *CYP2D6* gene.^34,35^ Respiratory depression and fatalities have been reported in postoperative paediatric patients and other settings, linked to *CYP2D6* ultra-rapid metaboliser status.^36,37^ Additionally, drug safety alerts from the Medicines and Healthcare products Regulatory Agency have highlighted an increased risk of Stevens–Johnson syndrome with carbamazepine, commonly prescribed for neuropathic pain, due to rare variants in *HLA-A* and *HLA-B* genes.^38^

The PROGRESS (Pharmacogenetics Roll Out – Gauging Response to Service) project, run by the NHS North West Genomic Medicine Service Alliance, is currently investigating the feasibility and health economic impact of genotyping common pharmacogenes in a primary care setting, including *CYP2C19* and *CYP2D6*.^39^ One of the aims of the project is to consider how results may be integrated into electronic health records and shared with patients for future reference.

### Antimicrobials

Genomics has a key role to play in combating antimicrobial resistance. In January 2024, the UK Health Security Agency published its five-year pathogen genomics strategy, highlighting the ability of genomic testing to swiftly identify pathogens, track transmission and predict sensitivities to antimicrobials.^40^ Together with host immunity profiling, this is being piloted for patients presenting with acute severe infection via an NHS Genomic Network of Excellence.^41^

## Whole-genome sequencing and brain tumours

The 2021 World Health Organization’s classification of tumours of the central nervous system adopted genomic alterations to define several new pathological entities in the brain for the first time.^42^ Alongside the continued development of precision therapies for cancer, this has synergised the requirement for genomic biomarkers to be identified in ‘real time’.

Multiple methods for genome sequencing currently exist, with panel-based approaches (incorporating a predefined set of known cancer-relevant mutations) predominating in the UK. These are often employed in conjunction with the piecemeal development of local paradigms to identify the pre-specified alteration using the cheapest and most feasible method (e.g. 1p19q codeletion identified through fluorescence in situ hybridisation for oligodendroglioma).

Whole-genome sequencing (WGS) is an alternate approach that provides untargeted coverage across the genome. Illumina-based short-read sequencing is the technique used most commonly and provides high-fidelity data at base pair resolution by sequencing DNA that has been fragmented and amplified in a massively parallel fashion. Over the past decade, novel approaches have been developed that sequence unfragmented DNA in its nascent or amplified form. These long-read technologies include the Oxford Nanopore and PacBio systems, with the former being tested in the NHS through a collaboration with Genomics England. In its nascent mode, long-read technology has the capacity to also provide information on epigenomic structures across the genome (e.g. DNA methylation), which may be of relevance in specific circumstances.

At present, WGS is available for several indications, including adult and paediatric brain tumours, via the national genomic test directory,^13^ specifically for short-read WGS. Interestingly, despite this availability, there has not been universal uptake of the service, with multiple barriers having been identified.

Several initiatives across the UK have sought to identify and overcome the barriers to WGS in adult brain cancer, which will likely be relevant to other cancers should WGS be adopted more widely. From a practical perspective, the predominant issue seems to be that of tissue collection and processing. The need for fresh-frozen tissue and tissue transfer to the local genomic laboratory hubs (of which 7 exist across England) has not been funded adequately. The additional consent form required for germline DNA analysis (identifying hereditary cancer risk) and addition to the Genomics England database are also problematic. Moreover, analysis of WGS data in the genomic laboratory hubs has been of low priority, with panel-based and historical approaches being prioritised for pragmatic reasons.

The ‘why order’ question has been a major issue in brain tumour WGS. With most adult brain tumours being diagnosed using common assays (e.g. histology and immunohistochemistry), the use of WGS and long-read sequencing likely has predominantly diagnostic utility in rarer tumour subtypes through identification of rarer mutations/gene fusions or using methylation classifiers (e.g. Heidelberg classifier for brain tumours).^43^

Importantly, none of these barriers are insurmountable and considerable effort is being exerted in understanding where small pockets of targeted funding (such as for the provision of genomics coordinators or even just an additional freezer) can be deployed. Discussion of data at genomic tumour advisory board meetings, providing additional layers of interpretation prior to feedback at regular multidisciplinary team meetings, is gaining traction as a model for rapid clinical data feedback.

The designation of glioblastoma (primary brain cancer) as a cancer of unmet need by Cancer Research UK indicates the lack of therapeutic progress in this field. A hope among the brain cancer community is that WGS will represent the route in to targeted precision medicine clinical trials.^44^ An adaptive precision platform study, recently funded by the Minderoo Foundation and Cancer Research UK, will seek to use real-time WGS in glioblastoma to target novel therapies. Importantly, the WGS data will not only provide the method for biomarker-based trial arm stratification but will also enable trial adaptation and post hoc analysis to better match patients with drug.

WGS has the potential to transform our treatment of patients but logistic challenges for rapid turnaround times exist. Definition of the most appropriate use case will likely be cancer specific but we predict that for brain tumours, shifting the view of WGS from a diagnostic to a treatment stratifying tool will be vital for improving prognosis in this group of patients who currently have very few treatment options and a mortality rate of 100%.

## Mainstreaming

The diagnostic tests for cancers are becoming more complex as our understanding of tumour molecular biology increases with the goal of improving patient outcomes. Precision medicine can be transformational, and utilises genetic, environmental and lifestyle factors to personalise strategies for treatments and to prevent disease. This field of medicine is advancing at pace, allowing healthcare professionals to personalise treatments, as in the previous examples of chemotherapy, immunotherapy and cancer vaccines.

Nevertheless, it is not just oncologists who are using genomics to tailor their approach. Surgeons can use genomic testing to guide and counsel their patients, for example when breast surgeons are considering removing the contralateral, unaffected breast in a woman with a known high-risk inherited gene alteration like *BRCA* or when colorectal surgeons may consider more extensive surgery in a patient with Lynch syndrome. These tests can also have an impact for relatives of the patients who are treated. Family members may elect (with appropriate counselling) to have genetic testing and consider risk-reducing surgery if they have inherited high-risk cancer predisposition genes. Patients with Lynch syndrome may also consider pharmacological risk-reducing strategies such as aspirin to reduce the risk of developing colorectal cancer by 50% (CAPP2 trial).^45^

Molecular (genomic) testing is developing into a diagnostic point-of-care test as results are required by the cancer multidisciplinary team to plan first-line and ongoing treatment. Historically, appropriate patients have been referred to clinical genetics teams for counselling and further testing but the waiting times for this can be too long, especially in a cancer treatment pathway. A solution to achieving timely testing is to offer pre-test genomic counselling and testing by an appropriately trained member of the cancer team, known as mainstreaming*.*^46,47^

**Figure 3:**
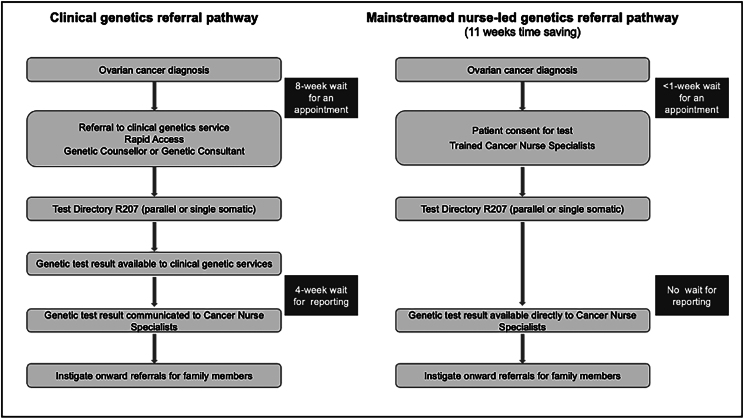
Comparison of the classical clinical genetics referral pathway and the new mainstreamed nurse-led pathway for ovarian cancer

A systematic review of the mainstreaming principles demonstrated the feasibility and utility of this approach.^48^ There were additional benefits of improving equity of access, faster genomic diagnosis and identifying risk of future cancers, with potential to also identify relatives at risk. Figure 3 illustrates the time savings/advantages of a mainstreamed pathway over the classical referral pathway. However, cascade testing of relatives remains under the auspices of the clinical genetic team. Patients report satisfaction in the continuity of care, trust and rapport they have developed with their cancer team, who can guide them through the genomic diagnostic testing and impact on their treatment. Any healthcare professional can be trained to provide the testing, with cancer nurse specialists evolving as the most likely team member owing to their patient pathway overview.^49^ The review suggests that the key to implementing successful mainstreaming involves job planning (time to provide mainstream consult and return of results), education and training, and pathway planning.^48^

NHS England’s seven Genomic Medicine Service Alliances will help embed the principle of mainstreaming and educate NHS staff.^50^ The NHS has launched transformation projects on Lynch syndrome, *BRCA* and *DPYD* as well as others focusing initially on colorectal, endometrial, ovarian, breast and prostate cancer.

Led by the Genomics Education Programme, in collaboration with the Genomic Medicine Service and the Academy of Medical Royal Colleges, the Clinical Pathway Initiative (CPI) has the aim of integrating genomic competences into the education and training of the NHS workforce, with a nationally coordinated and consistent approach.^51^ These CPI projects are being developed with relevant tumour site clinicians to identify where in the diagnostic pathway testing is required, and what the education and training needs of the clinicians are, with the necessary supporting documents (patient information leaflets, request forms, process mapped pathways) being included in ‘mainstreaming toolkits’, all available on the NHS England website.^51^

## Conclusions

The evolving landscape of cancer treatment demands a dynamic and integrated approach from surgeons and the multidisciplinary team. Immunotherapy, ctDNA, pharmacogenomics, cancer vaccines, mainstreaming and WGS are just some of the innovations that have the potential to redefine the standards of care. They offer new hope and improved outcomes for patients undergoing curative cancer surgery. The continued exploration of these innovative diagnostics and therapies, the genomic pathway evolution and their application in diverse cancer types highlights the transformative impact of precision medicine in the field of surgery.
